# Right Heart Structure, Geometry and Function Assessed by Echocardiography in 6-Year-Old Children Born Extremely Preterm—A Population-Based Cohort Study

**DOI:** 10.3390/jcm10010122

**Published:** 2020-12-31

**Authors:** Lilly-Ann Mohlkert, Jenny Hallberg, Olof Broberg, Gunnar Sjöberg, Annika Rydberg, Petru Liuba, Vineta Fellman, Magnus Domellöf, Mikael Norman, Cecilia Pegelow Halvorsen

**Affiliations:** 1 Department of Clinical Science, Intervention and Technology, Division of Pediatrics, Karolinska Institutet, 141 52 Stockholm, Sweden; mikael.norman@ki.se; 2 Department of Pediatric Cardiology, Sachs’ Children and Youth Hospital, Södersjukhuset, 118 83 Stockholm, Sweden; jenny.hallberg@ki.se (J.H.); cecilia.pegelow-halvorsen@sll.se (C.P.H.); 3 Department of Clinical Science and Education, Karolinska Institutet, 118 83 Stockholm, Sweden; 4 Department of Clinical Sciences, Division of Pediatric Cardiology, Lund University, and Pediatric Heart Center, Skåne University Hospital, 221 00 Lund, Sweden; olof.broberg@med.lu.se (O.B.); petru.liuba@med.lu.se (P.L.); 5 Department of Women’s and Children’s Health, Karolinska Institutet, 171 77 Stockholm, Sweden; gunnar.sjoberg@sll.se; 6 Department of Clinical Sciences, Pediatrics, Umeå University, 901 87 Umeå, Sweden; annika.rydberg@umu.se (A.R.); magnus.domellof@umu.se (M.D.); 7 Children’s Hospital, University of Helsinki, 00014 Helsinki, Finland; vineta.fellman@med.lu.se; 8 Department of Clinical Sciences, Division of Pediatrics, Lund University, Skåne University Hospital, 221 00 Lund, Sweden; 9 Department of Neonatal Medicine, Karolinska University Hospital, 141 86 Stockholm, Sweden

**Keywords:** echocardiography, myocardial performance index, patent ductus arteriosus, preterm infant, pulmonary arterial hypertension, right atrium, right ventricle

## Abstract

Preterm birth has been associated with altered cardiac phenotype in adults. Our aim was to test the hypothesis that children surviving extremely preterm birth have important structural or functional changes of the right heart or pulmonary circulation. We also examined relations between birth size, gestational age, neonatal diagnoses of bronchopulmonary dysplasia (BPD) and patent ductus arteriosus (PDA) with cardiac outcomes. We assessed a population-based cohort of children born in Sweden before 27 weeks of gestation with echocardiography at 6.5 years of age (*n* = 176). Each preterm child was matched to a healthy control child born at term. Children born preterm had significantly smaller right atria, right ventricles with smaller widths, higher relative wall thickness and higher estimated pulmonary vascular resistance (PVR) than controls. In preterm children, PVR and right ventricular myocardial performance index (RVmpi’) were significantly higher in those with a PDA as neonates than in those without PDA, but no such associations were found with BPD. In conclusion, children born extremely preterm exhibit higher estimated PVR, altered right heart structure and function compared with children born at term.

## 1. Introduction

Although preterm birth is still a major cause of early mortality worldwide, infants born preterm are most likely to survive in high and middle resource settings [1,2]. During the neonatal period, extremely preterm infants (born before 28th gestational week) often have respiratory and circulatory disorders. While acute problems usually resolve over time, there are several follow-up studies demonstrating reduced lung function and altered cardiovascular development in children, adolescents and adults surviving preterm birth [3,4,5,6]. This may have implications for the increased risks of chronic obstructive lung disease, systemic and pulmonary hypertension, and cardiovascular disease that have been reported in adults born preterm [7,8].

Two comprehensive evaluations of circulation in young adults born preterm have pointed at the right heart and the pulmonary circulation as particularly exposed to long-standing cardiovascular changes. Lewandowski et al. found that the right ventricular (RV) cavity of young adults born preterm at a mean gestational age (GA) 30 weeks, was 8–10% smaller and the RV mass 20% larger than in same-aged controls born at term. These changes showed an inverse dose-response relationship with GA [6]. In another study, healthy young adults born before 32 gestational weeks and undergoing cardiac catheterization were found to have elevated pulmonary arterial pressure, stiffer pulmonary vascular bed and right ventricular dysfunction [8]. More recently, a strong association between preterm birth and increased risk of pulmonary arterial hypertension (PAH) has been reported from a registry study [7]. Whether or not these circulatory alterations are already expressed in early childhood is poorly explored.

The primary aim of this study was to examine if children surviving extremely preterm birth at an age of 6.5 years exhibit significant structural or functional changes of pulmonary circulation or right side of the heart. Secondary aims included to study any relations between perinatal exposures such as birth size, gestational age and neonatal conditions affecting pulmonary circulation, and right heart outcome measurements in pre-school children.

## 2. Methods

### 2.1. Participants

The participants were recruited from the prospective national population-based cohort EXPRESS (Extremely Preterm infants in Sweden Study) including all infants born in Sweden before 27 weeks of gestation from 1 April 2004 to 31 March 2007. In 2010–2013, all EXPRESS children were invited to a comprehensive follow up at 6.5 years (±3 months), and in three out of six regions (Lund, Stockholm and Umeå), cardiovascular and lung function assessments were performed. In these three regions, 250/494 (51%) of the survivors of the original EXPRESS cohort were residing. In total, 176/250 (70% follow-up rate) EXPRESS children accepted the invitation. Drop-out analysis, i.e., of those that declined participation (*n* = 40), were lost to follow-up (*n* = 7), or passed date for latest inclusion (*n* = 27), did not disclose any significant differences in GA, birth weight or sex distribution between those EXPRESS children participating in the study and those lost to follow-up, for details please see previous publications [9,10]. Using the Swedish Medical Birth Register held at the National Board of Health and Welfare, each child born extremely preterm (EXPT) was matched to a healthy control child born at term (CTRL) with same sex, date of birth, birth hospital, residency and mother’s country of birth. Because of limited inclusion and analysis capacity of one region (Lund), only the EXPT children from that site had right heart and pulmonary circulation investigated. Altogether, 176 EXPT and 134 CTRL children were assessed.

Inclusion and exclusion criteria for EXPT, perinatal characteristics, data on survival, neonatal morbidity, neurodevelopmental outcomes at 30 months and 6.5 years-of-age, as well as details on lung function, vascular assessments and left ventricular functions in childhood have previously been reported [2,3,9,10,11,12,13,14,15,16,17]. Twelve EXPT children were excluded due to congenital heart malformations, and CTRL children were only invited if they had a history of a normal neonatal period without any diagnoses of congenital malformations or ongoing cardiovascular or pulmonary disease.

### 2.2. Ethics

All parents and children received oral and written information, and the parents or legal guardians to participating children signed informed consent. Study approval was obtained from the Regional Ethics Review Board in Stockholm (no. 2010/520-31/2, amendment no.2011/376-32).

### 2.3. Clinical and Cardiac Assessments

Previous medical history of participants and parents, including maternal smoking during pregnancy, was obtained using a questionnaire and the results have been reported elsewhere [9,10].

Small for gestational age (SGA) was defined as birthweight below two standard deviations from the predicted mean birthweight for GA and sex, according to Swedish standards for fetal growth [18]. A hemodynamically significant patent ductus arteriosus (PDA) during the neonatal period was defined as a PDA in need of any treatment (pharmacologically or surgically). Bronchopulmonary dysplasia (BPD) was defined as need of extra oxygen supply at 36 weeks of postmenstrual age, and severe BPD was defined as oxygen need of at least 30% or positive pressure ventilation at 36 weeks of postmenstrual age [3].

Before echocardiographic examination, height and weight were measured. BMI was calculated as weight/height^2^ and body surface area (BSA) was calculated according to Haycock [19]. A validated oscillometric device, Omron HEM 907 (Omron Healthcare, Kyoto, Japan) was used to measure heart rate, systolic (SBP) and diastolic (DBP) blood pressures. Results from vascular assessments have been reported earlier [17].

The echocardiographic equipment was Acuson SC2000^TM^ (Siemens Medical Solutions, Mountain View, CA, USA) with a multi-frequency 8–3 MHz vector wide-view array transducer in Stockholm. In Lund and Umeå, Philips iE33 (Philips Healthcare, Amsterdam, The Netherlands) was used with a multi-frequency phased array transducer. A complete echocardiographic assessment was performed to exclude structural heart defects.

### 2.4. Determination of Right Heart Outcome Variables

**Figure 1:**
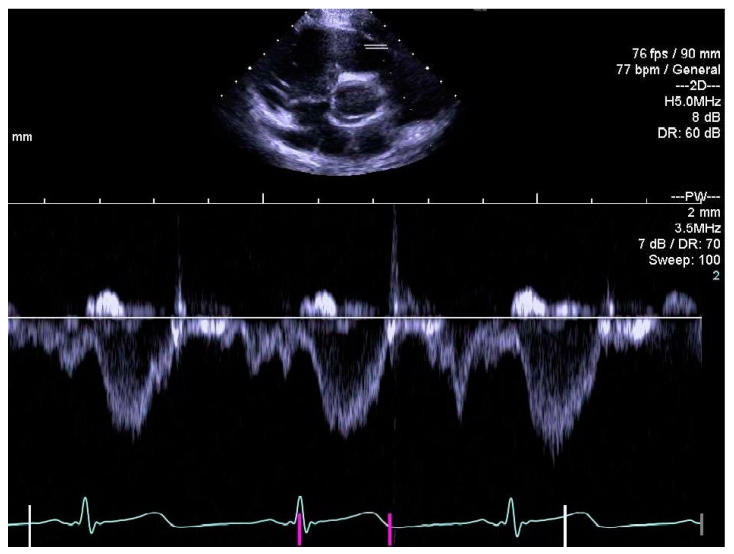
Pulsed wave Doppler of right ventricular outflow tract (RVOT) for measurement of velocity time integral, RVOTvti.

**Figure 2:**
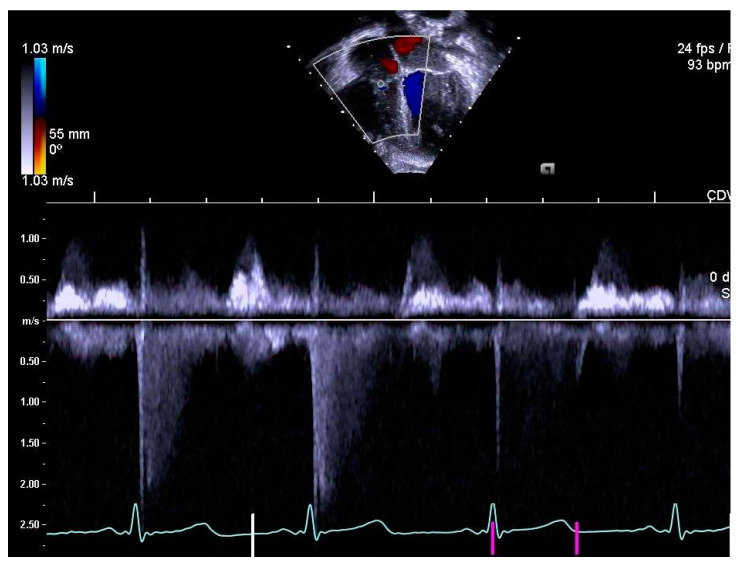
Tricuspid regurgitation (TR) measured by continuous wave Doppler.

**Figure 3:**
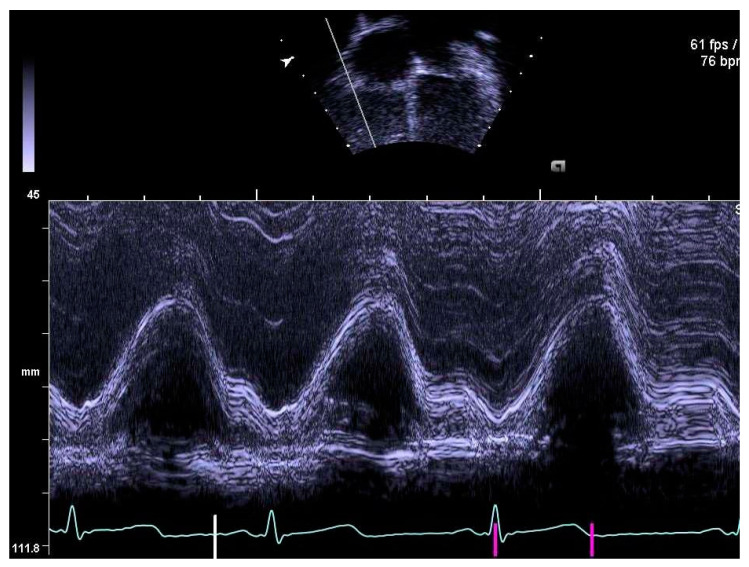
Tricuspid annular plane systolic excursion, TAPSE, measured by M-mode.

**Figure 4:**
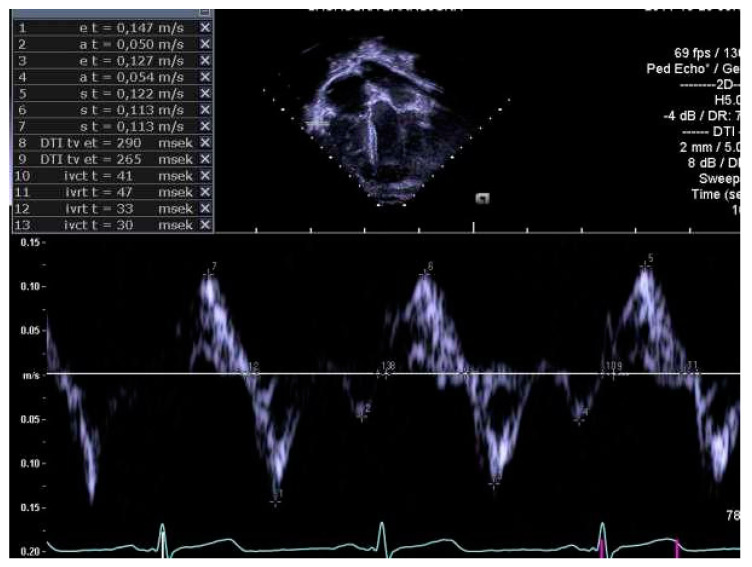
Right ventricular myocardial performance index (RVmpi’) assessed by pulsed wave tissue Doppler of right ventricular free wall.

The same experienced cardiac sonographer at each centre investigated all participants and followed a predefined standardized protocol. Image analyses of the right heart and the pulmonary arteries, as well as systolic and diastolic functions acquired with 2D echocardiography and blood- and tissue Doppler, were performed off-line by two operators: one (LAM) for recordings from Stockholm and Umeå, and another (OB) for Lund. Both operators were blinded to group belonging, and vascular dimensions as well as systolic and diastolic functions were determined using similar techniques as in previously reports of the systemic arteries and left heart [9,10]. RV volumes were calculated with velocity time integral for right ventricular outflow tract (RVOTvti) estimated by pulsed wave Doppler (PW) and pulmonary valve annulus diameter (PVann), Figure 1. RV relative wall thickness (RWT) was calculated as (anterior wall + interventricular septum)/ RV width. Sphericity index (SI) was calculated as diameters for length/width for the atrium and ventricle, respectively. Pulmonary vascular resistance (PVR) was estimated by Doppler, calculating the ratio using the simplified formula described by Abbas et al. 2003, for tricuspid valve regurgitation (TR), Figure 2, divided by RVOTvti [20]. Systolic function was evaluated by tricuspid annular plane systolic excursion (TAPSE), Figure 3 and diastolic function was assessed by early (E) and late (A) velocities of the tricuspid valve with pulsed wave and, with tissue Doppler (s’, e’, a’). To estimate RV filling pressure, the E/e’ ratio was calculated. Systolic ejection time (et), isovolumic relaxation time (ivrt) and isovolumic contraction time (ivct) were assessed with tissue Doppler to calculate right ventricular myocardial performance index (RVmpi’), using the formula (ivrt + ivct)/et, Figure 4.

### 2.5. Statistical Analyses

We performed statistical analyses using STATA^®^ 16C (StataCorp LLC, College Station, TX, USA). Results are reported as means and standard deviations (SD) or numbers (proportions and percentages). The distributions of outcome variables were visually inspected and based on summary statistics of the software comparing means and medians, standard deviations, and percentiles, and providing skewness, we assumed all variables to be normally distributed. The study was powered to detect small to moderate effect sizes of 0.3 SD or more and a *p*-value < 0.05 was considered statistically significant. To compare groups, Student’s t-test or Chi-squared test were used. We adjusted mean differences of right heart dimensions and volumes and calculated 95% confidence intervals using multiple linear regression including group belonging (EXPT or CTRL), BSA and site as independent variables. Functional outcome variables such as velocities and time intervals were only adjusted for site.

After analysing group differences between EXPT and CTRL children, we tested for differences within the EXPT group. These analyses tested whether gestational age (22–24 weeks versus 25–26 weeks), SGA, a diagnosis of PDA or of severe BPD during the neonatal period, were associated with right heart outcomes at 6.5 years.

## 3. Results

### 3.1. Cohort Characteristics

Maternal age and rates of family history of cardiovascular disease did not differ between the EXPT and CTRL groups, whereas the proportion of university-educated mothers was lower in EXPT than in CTRL. There were no group differences in participants’ age or sex, but weight, height and BSA were lower in EXPT than in CTRL children. Systolic blood pressure was similar whereas diastolic blood pressure and heart rate were higher in EXPT than in CTRL, Table 1. Two children in the EXPT group were excluded from further analyses because of previously undiagnosed left ventricular outflow tract obstruction (*n* = 1) and bilateral pulmonary branch stenoses (*n* = 1), respectively.

### 3.2. Completeness of Image Analysis

We were able to analyze 87–96% of the outcome variables for right ventricle and atrium, 69–84% of pulmonary artery dimensions and, 85–95% of systolic and diastolic measurements by the criteria set in international standards and guidelines for echocardiographic assessments of children [21]. All assessments were reviewed prior to analysis according to the predefined standard operation protocol. Images that did not meet these criteria were not accepted for analysis and were registered as missing in the dataset. Four children agreed to weight and height measurements but declined echocardiographic assessments. The most common cause of incomplete echocardiographic data acquisition was the participants’ inability to cooperate to a full investigation (one hour).

### 3.3. Right Heart Dimensions, Myocardial Thickness and Volumes

EXPT children had smaller right ventricular (RV) and right atrial (RA) widths than CTRL children. RA length was also smaller in EXPT than in CTRL. There was no significantly difference of RV length after adjustment for BSA, whereas the RV width was smaller in EXPT than in controls. The shape of the right heart also differed between the groups: the RV sphericity index (length/width) among EXPT participants was higher than in CTRL, Table 2.

RV thickness of the anterior wall did not differ between the two groups. The interventricular septum was thinner in EXPT than in CTRL children, but the difference disappeared after adjustment for BSA. Moreover, the RV relative wall thickness was higher in EXPT than in CTRL. RV stroke volume (SV) was 6% smaller in EXPT than in CTRL and despite a slightly higher heart rate, cardiac output (CO) was lower in EXPT than in CTRL. However, this group difference disappeared after adjustment for BSA, Table 2. Intraobserver variability expressed as coefficient of variation for echocardiographic assessments of two-dimensional RV length was 4.1%.

### 3.4. Pulmonary Artery Diameters

Pulmonary artery diameters were significantly smaller in EXPT than in CTRL but after adjustment for BSA, these differences were no longer significant, Table 2.

### 3.5. Right Heart Systolic and Diastolic Functions

Systolic function expressed as TAPSE was lower in EXPT compared with CTRL after adjustment. RV myocardial systolic velocity (s’) of the free wall and interventricular septum showed no differences between EXPT and CTRL. Adjusted RVmpi’ of the free wall assessed with tissue Doppler was slightly lower in EXPT than in CTRL.

PVR was significantly higher in EXPT than in CTRL and according to the limit of TR/RVOTvti > 0.2 for PVR > 2 Woods units (WU), 32% of EXPT children and 16% of CTRL children exceeded this limit [21]. However, one EXPT child and one CTRL child had an indication of pulmonary arterial hypertension > 6 WU, i.e., a ratio of tricuspid regurgitation to velocity time integral of right ventricular outflow above the cut-off of 0.275 [22].

Except for shorter myocardial isovolumic contraction times (ivct) of the septum and the free wall in EXPT than in CTRL, there were no statistically significant differences in diastolic function between the two groups, Table 3. Intraobserver variability expressed as coefficient of variation for echocardiographic assessments of pulsed Doppler RVOTvti was 5.2%.

### 3.6. Perinatal Risk Factors and Right Heart Structure and Function in Children Born Extremely Preterm

When comparing EXPT children born SGA (*n* = 27) with those born appropriate for gestational age (*n* = 142), we found no statistically significant differences in right heart dimensions, myocardial thickness, and volumes, systolic and diastolic myocardial functions, or PVR, supplemental Tables S1 and S2. The same was found for comparisons between children born at 22–24 weeks (*n* = 50) and at 25–26 weeks of GA (*n* = 119) with three exceptions: children born at 22–24 weeks had a slightly higher RV sphericity index, 0.4 cm/s lower right ventricular myocardial systolic velocity (TVs’) and, 0.4 cm/s lower diastolic velocity (TVa’) than children born at 25–26 weeks of GA, supplemental Tables S3 and S4.

Children born extremely preterm with a hemodynamically significant neonatal PDA (*n* = 106), exhibited a higher PVR compared with those without PDA (*n* = 66), (0.189 (0.04) versus 0.170 (0.04); *p* = 0.03). Children with PDA also had a higher myocardial performance index of the free right ventricular wall (0.37 (0.1) versus 0.33 (0.1); *p* = 0.036). In addition, the PDA group had a lower stroke volume (14.8 (2.9) versus 16.0 (3.6) ml; *p* = 0.040), but this difference was no longer statistically significant after adjusting for BSA. There were no other statistically significant differences in right heart dimensions, myocardial thickness or volumes between children with and without neonatal PDA, Table 4 and Table 5.

Children diagnosed with severe BPD (*n* = 29), had smaller RV width and higher sphericity index than those without severe BPD (*n* = 131), Table 6. There were no other statistically significant differences in right heart dimensions, systolic or diastolic functions, or in PVR between children according to history of severe BPD, Table 7.

All EXPT children had a sex matched CTRL, supplemental Tables S5–S8, but by per-forming multiple regression analyses we found that within EXPT group girls exhibited significantly larger RA and RV width than boys, whereas among CTRL children that rela-tion was inverse (data shown on request).

## 4. Discussion

This population-based cohort study of right heart structure, shape and function demonstrated that children born before the 27th gestational week had significantly smaller right atria, smaller RV width, higher echocardiographic estimated pulmonary vascular resistance, and reduced RV systolic globular function compared to term born controls. A more globe-shaped left ventricle has previously been found in children and adults born preterm [10,23]. The current data showing a higher RV sphericity index in EXPT than in CTRL extend these findings of an altered cardiac phenotype after preterm birth [24].

One of our main objectives was to explore if healthy children born extremely preterm had early signs of pulmonary hypertension. To do so, we assessed the size of the RA because individuals with pulmonary hypertension often express RA enlargement due to tricuspid regurgitation and increased right ventricular pressure. However, we found only 2/250 children with an indication of pulmonary hypertension and the RA were actually significantly smaller in EXPT than in controls.

Young adults born extremely preterm have been reported to face an increased risk of heart failure, and cardiomyopathy has been suggested as an underlying cause [24]. The RV sphericity index can be used to assess early signs of cardiomyopathy because a globe shaped RV would indicate volume overload and increased pressure. Within EXPT children, there were no signs of a globe shaped RV, but RV sphericity index was significantly higher in EXPT than in CTRL. This could be an early sign of altered RV performance. In addition, the RV may be adapting to the more globe shaped LV found in EXPT-subjects in earlier studies of this and other preterm cohorts [10,25].

Pulmonary vascular resistance was higher in EXPT children than in CTRL. PVR is determined by the microvascular density and the tone of precapillary resistance vessels in the lung. In line with our findings of an increased PVR, lung blood flow has been reported to be significantly decreased in children born preterm and in adult sheep born preterm [26,27]. Given a reduced lung blood flow, it would be expected that the pulmonary artery diameters were smaller in EXPT than in controls, but they were not. A suggestion for an explanation is the previously reported elevated distending pressure of the pulmonary artery in children born preterm [8].

Unfortunately, echocardiography is not the method of choice when assessing right ventricular mass and therefore we could not report such data. However, the thickness of the RV anterior wall and of the IVS could be assessed and did not differ in EXPT compared with CTRL. The lack of a group difference in thickness of the RV anterior wall or IVS may be due to the early age of the participants of this study. In adult sheep born preterm, the RV wall has been reported to be thinner, with a reduction in the number and size of heart muscle cells compared with controls born at term [27]. RV RWT has been associated with RV remodeling predicting long-term outcome in patients with pulmonary hypertension [28]. Wall thickness did not differ, but RV RWT was higher in EXPT compared with CTRL.

We have previously found that the mass of the left ventricle was reduced in EXPT compared with CTRL, also after adjustment for BSA [10]. This is in line with recent MRT data from adolescents and young adults born preterm in which both the LV and RV were reported to have lower mass than controls of similar age born at term [29]. In contrast, adult MRT studies showed increased mass of both the left and right ventricle in subjects born preterm [6,23]. An important implication of these findings is that, albeit a higher systemic and pulmonary vascular resistance [17,30], LV or RV myocardial hypertrophy may not be evident in preterm children before school-start, warranting longer follow-ups. Another interpretation is that cohort selections may differ between the studies.

TAPSE was lower in EXPT than in controls, but there was no group difference in TV free wall s’ and RVmpi’ was lower in EXPT than in controls. A potential explanation for these findings could be that TAPSE is easy to measure and suggested to be a more sensitive estimate than RVmpi’ and RV free wall s’ when evaluating RV systolic function [21]. Moreover, our findings of smaller RA and lower RVmpi’ in EXPT could reflect that the EXPT infants were assessed at an early age before pulmonary hypertension had developed.

Early remodeling of the myocardium rarely seems to be of clinical concern during early childhood. Fetal adaptations to intrauterine circulatory stress in the last trimester rarely result in heart failure or hydrops. Although there used to be a common opinion about RV being inferior to the development of the LV, this has been revised and is now thought to be a matter of ventricular interdependency [31]. RV systolic function is mediated by longitudinal muscle fibers and is therefore more sensitive to volume or pressure overload than LV [32]. We did not find any significant differences in the myocardial performance indices of septal or free RV wall. However, an early alteration of RV systolic function could not be ruled out with TAPSE being significantly lower in the EXPT group. This is consistent with findings recently reported in a multimodal RV study of moderately preterm born adults [33].

PDA is common in extremely preterm infants and affected 60% in this study. Breatnach et al. reported no differences in right ventricular systolic function with tissue Doppler (TVs’) and tricuspid annular plane systolic excursion (TAPSE), but a lower fractional area change and lower basal systolic longitudinal strain estimated with speckle tracking echocardiography in preterm infants with PDA than in infants without PDA [34]. However, in another longitudinal study in infancy, no such associations were found [35]. In this study, EXPT children with a hemodynamically significant PDA in the neonatal period showed a significantly higher RVmpi’ of the free wall compared with EXPT without PDA. This is consistent with the finding of a higher PVR in EXPT children treated for PDA in the neonatal period and could be an indication of increased RV workload. The RVmpi’ of the septal wall did not differ in relation to neonatal PDA, most likely as it represents the pressure and workload of the LV to a higher degree than RVmpi’ of the free wall.

Invasively assessed hemodynamics in children with pulmonary hypertension showed that they had increased RVmpi’ as derived by tissue Doppler imaging [36]. Although echocardiographically estimated RVmpi’ has to be interpreted with caution, studies of adults with chronic thromboembolic pulmonary hypertension before and after thromboendarterectomy, showed that RVmpi’ was related to PVR measured with right heart catheterization [37]. Based on these reports, it can be assumed that the higher RVmpi’ found in children treated for PDA as neonates are in the upper normal range, although pediatric reference values of RVmpi’ are missing. Moreover, the finding of a higher PVR in EXPT treated for PDA in the neonatal period, could be a reason for a targeted follow-up of these children.

A history of BPD has been associated with reduced lung function at follow-up, and is also suggested to influence right ventricular geometry and mass in young adults [5,6]. In a previous publication, we did not find any association between lung volumes and pulmonary vascular resistance in extremely preterm children [16]. This is consistent with the findings herein and by others [27] of no association between severe BPD and right heart dimensions or pulmonary vascular resistance, but inconsistent with a meta-analysis reporting BPD severity as a risk factor for pulmonary hypertension [38]. However, the follow-up time in the studies included in that meta-analysis was only a few months [38]. Therefore, factors other than neonatal lung disease may be more important for the development of pulmonary arterial hypertension after preterm birth over time [4,39].

By using sex-matched controls, we adjusted our data for sex differences, but found an inverse relation of sizes of atrial and ventricular widths among each group. CTRL boys had larger sizes compared with CTRL girls even after adjustment for BSA. In the EXPT group there was an inverse relation. This may be an indication of males being more vulnerable to hemodynamic changes than females in early life, an important finding to be investigated further.

Recent genetic studies have found common genomic variants in women with a history of delivering preterm [40]. Women delivering preterm are also at higher risk of developing hypertension or cardiovascular disease in later life [31]. Most likely, cardiovascular outcomes after preterm birth could be attributed to both developmental programming and genetic factors. Irrespective of origins, the present examinations of children at pre-school age have revealed that phenotypic alterations occur early.

A strength of this study is the population-based prospective design and a high survival rate (78%) contributing to the sample size with enough power to detect small effect sizes. All participants underwent a complete echocardiographic examination with findings within normal range. There was only one operator at each site performing the echocardiographies, and two of them conducted the off-line analyses, blinded to group belonging. EXPT and CTRL children were only assessed if healthy, thus avoiding any contributions from acute infectious disease to our results. Measurements were completely non-invasive and therefore acceptable to healthy, small children.

Limitations of this study include dropouts, incompleteness of image analysis and common pitfalls using echocardiography such as angle dependent measurements and inter-operator variability. The gold standard for estimation of pulmonary vascular resistance is measurement by invasive heart catherization. We refrained MRT and invasive measurements by ethical reasons, according to anesthesia requirements for the ability to investigate children at pre-school age. Speckle tracking echocardiography could not be performed in all three centers, and therefore the RV strain was not included in our analyses. Another limitation is that we did not examine the parents with echocardiography or blood pressure. However, multiple regression analysis did not reveal any contributions to our outcomes from a family history of cardiovascular disease. In the EXPRESS database, there is no information from neonatal echocardiographies, and, therefore, it was not possible to evaluate echocardiographic assessments longitudinally.

### Conclusions

Children born extremely preterm have smaller right heart atria, altered right ventricular shape and higher estimated pulmonary vascular resistance compared with term born children at 6.5 years. A patent ductus arteriosus in need of treatment during neonatal period may have contributed to these cardiac alterations. Reassuringly, there were no signs of clinically significant cardiac problems at pre-school age in children born extremely preterm, and therefore routine echocardiographic assessment of asymptomatic pre-school children born extremely preterm may not be necessary. If circulatory or pulmonary symptoms should continue after the neonatal follow-up or arise later, however, we recommend a diagnostic workup including echocardiography. In such assessments, estimated PVR (calculated by tricuspid regurgitation and RVOTvti), TAPSE and RV relative wall thickness are suggested to be included in addition to standard echo measurements. To determine the longer-term significance of our findings, circulatory research in older survivors of extremely preterm birth is warranted

## Figures and Tables

**Table 1:** Characteristics of 6.5-year-old children born extremely preterm (EXPT) and in controls born at term (CTRL).

	EXPT (*n* = 176)	CTRL (*n* = 134)	*p*-Value
**Maternal data**			
Age, years (mean and range)	31.4 (18–46)	31.8 (21–43)	0.47
University education	82 (47%)	84 (63%)	0.004
Family history of CVD ^a^	128 (74%)	102 (77%)	0.65
**Pregnancy data**			
Maternal smoking	9 (5%)	2 (1%)	0.09
Preeclampsia	16 (10%)	0	NA
Multiple birth	29 (17%)	0	NA
**Neonatal data**			
Gestational age, weeks Range	24.9 (1.0) 22–26	39.4 (1.2) 37–41	NA
Boys	98 (55%)	79 (60%)	0.53
Birth weight, g Range	788 (169) 348–1161	3591 (461) 2430–4315	NA
SGA at birth	27 (16%)	0	NA
BPD moderate	131 (74%)	0	NA
BPD severe	29 (16%)	0	NA
PDA	106 (60%)	0	NA
Mechanical ventilation	148/171 (87%)	0	
Duration of mechanical ventilation, days Range	14.9 (15.5) (0–121)	0	NA
**6.5 year follow-up**			
Age, months	80.8 (2.3)	81.1 (2.0)	0.23
Weight, kg	20.6 (3.6)	24.3 (3.9)	<0.001
Height, cm	118.1 (5.6)	123.1 (5.0)	<0.001
BMI, kg/m^2^	14.7 (1.6)	16.0 (2.0)	<0.001
BSA, m^2^	0.82 (0.09)	0.91 (0.09)	<0.001
HR, bpm	88 (13)	85 (9)	0.016
SBP, mmHg	98 (8)	97 (8)	0.38
DBP, mmHg	57 (6)	55 (6)	0.005

Data are mean (SD) or numbers (%) if not indicated otherwise; ^a^ Family history of cardiovascular disease (CVD): myocardial infarction, stroke, or coronary by-pass surgery in first degree relatives of mother or father; BMI = body mass index, BPD = Bronchopulmonary dysplasia, BSA = body surface area, DBP = diastolic blood pressure, HR = heart rate, PDA = patent ductus arteriosus, SBP = systolic blood pressure, SGA = small-for-gestational age.

**Table 2:** Right heart dimensions and volumes in 6.5-year-old children born extremely preterm (EXPT) and in controls born at term (CTRL).

	Accepted for Analysis EXPT/CTRL	EXPT ^a^, *n* = 172	CTRL ^a^, *n* = 133	*p*-value	Adjusted Mean Difference ^b^, (95% CI)	*p*-Value
**RA dimensions**						
RA length	151/126	31.9 (3.4)	35.6 (3.4)	<0.001	−1.1 (−1.8;−0.3)	0.006
RA width	150/126	27.9 (3.1)	31.5 (3.2)	<0.001	−2.0 (−2.8;−1.2)	<0.001
RA SI	150/126	1.2 (0.1)	1.1 (0.1)	0.29	0.04 (0.008;0.08)	0.016
**RV dimensions**						
RV length	152/128	50.7 (4.5)	53.4 (4.1)	<0.001	−0.5 (−1.6;0.6)	0.34
RVwidth	152/129	27.2 (2.8)	29.4 (2.8)	<0.001	−1.7 (−2.4;−0.9)	<0.001
RV SI	152/128	1.9 (0.2)	1.8 (0.2)	0.04	0.09 (0.03;0.1)	0.002
LV/RV length	151/127	1.08 (0.06)	1.10 (0.06)	0.004	−0.02 (−0.04;−0.008)	0.004
**Wall thickness**						
RVAW	100/112	2.6 (0.7)	2.7 (0.8)	0.59	−0.007 (−0.2;0.2)	0.95
IVS	156/132	5.5 (0.9)	6.1 (0.8)	<0.001	−0.09 (−0.3;0.1)	0.39
RWT	95/109	0.32 (0.04)	0.30 (0.04)	0.012	0.02 (0.002;0.03)	0.026
**PA dimensions**						
PV ann	130/90	16.2 (2.2)	17.7 (1.8)	<0.001	−0.3 (−0.8;0.3)	0.33
MPA	141/125	17.0 (1.8)	18.0 (1.8)	0.001	−0.008 (−0.5;0.5)	0.97
LPA	120/112	10.4 (2.3)	12.0 (1.4)	<0.001	−0.2 (−0.6;0.2)	0.34
RPA	125/118	10.3 (2.2)	11.8 (1.4)	<0.001	−0.02 (−0.4;0.3)	0.89
**Volumes**						
SV, ml	117/85	15.2 (3.2)	17.0 (2.7)	0.0001	−1.1 (−2.0;−0.1)	0.026
CO, l/min	117/85	1.3 (0.3)	1.4 (0.2)	0.011	−0.07 (−0.2;0.02)	0.11

Data are expressed in mm if not indicated otherwise; ^a^ Crude value; ^b^ Mean difference adjusted for body surface area (m^2^) and site; CO = cardiac output, IVS = interventricular septae, PA = pulmonary artery, PVann = pulmonary artery valve annulus, MPA = main pulmonary artery, LPA = left pulmonary artery, RA = right atrium, RWT = relative wall thickness, RPA = right pulmonary artery, RV = right ventricle, RVAW = right ventricular anterior wall, SI = sphericity index, SV = stroke volume.

**Table 3:** Right heart systolic and diastolic function in 6.5-year-old children born extremely preterm (EXPT) and in controls born at term (CTRL).

	Accepted for Analysis EXPT/CTRL	EXPT ^a^ *n* = 172	CTRL ^a^ *n* = 133	*p*-Value	Adjusted Mean Difference ^b^, (95% CI)	*p*-Value
**Systolic function**						
TAPSE, mm	146/123	20.7 (2.9)	21.2 (2.7)	0.15	−0.8 (0.5;1.5) ^*^	0.037
TVs’septal, cm/s	143/114	6.7 (1.0)	6.6 (0.7)	0.53	0.1 (−0.4;0.1)	0.28
TVs’ free wall, cm/s	115/77	11.4 (2.2)	11.8 (1.7)	0.15	−0.5 (−1.1;0.1)	0.10
mpi’, septal	114/83	0.44 (0.07)	0.45 (0.07)	0.16	−0.01 (−0.04;009)	0.24
mpi’, free wall	132/110	0.35 (0.1)	0.34 (0.1)	0.18	−0.03 (−0.05;−0.003)	0.027
PVR	116/117	0.187 (0.04)	0.174 (0.03)	0.006	0.02 (0.01;0.03)	<0.001
TR, m/s	125/121	2.0 (0.1)	2.0 (0.3)	0.31	0.1 (0.04;0.2)	0.001
RVOTvti, cm	148/126	11.9 (2.4)	11.8 (1.8)	0.71	−0.5 (−1.0;−0.07)	0.024
**Diastolic function**						
TVE, cm/s	147/129	50.5 (11)	49.1 (10)	0.28	−1.0 (−3.4;1.5)	0.44
TVA, cm/s	139/126	32.6 (7.8)	32.0 (7.9)	0.56	−0.4 (−2.4;1.6)	0.68
**Septal**						
Annular e’, cm/s	143/113	12.4 (1.4)	12.1 (1.3)	0.13	0.09 (−0.3;0.5)	0.63
Annular a’, cm/s	143/114	4.5 (1.1)	4.5 (0.9)	0.45	−0.02 (−0.3;0.2)	0.91
E/e’	132/112	4.1 (0.9)	4.0 (1.0)	0.38	−0.09 (−0.3;0.2)	0.46
ivct, msec	143/113	60 (12)	65 (13)	0.001	−4.6 (−8.0;−1.3)	0.006
ivrt, msec	143/112	57 (10)	56 (8)	0.65	−0.7 (−2.9;1.6)	0.57
**Free wall**						
TVe’, cm/s	134/113	15.0 (2.7)	14.1 (2.1)	0.003	0.5 (−0.1;1.2)	0.13
TVa’, cm/s	132/113	7.6 (2.0)	6.8 (1.7)	0.003	0.5 (−0.01;1.0)	0.056
E/e’	124/112	3.4 (0.8)	3.5 (0.9)	0.23	−0.2 (−0.4;−0.002)	0.059
ivct, msec	133/113	59 (14)	65 (16)	0.002	−5.9 (−9.8;−2.0)	0.003
ivrt, msec	133/111	25 (13)	35 (21)	<0.001	−0.6 (−3.9;3.1)	0.72

^a^ Crude value. Values are presented as mean and SD; ^b^ Mean difference adjusted for site; * Mean difference adjusted for body surface area (m^2^) and site; E/e’ = transtricuspid early diastolic velocity/ tricuspid annular early diastolic velocity; ivct = isovolumic contraction time; ivrt = isovolumic relaxation time; MPI = myocardial performance index; PVR = estimated pulmonary vascular resistance (=TR/RVOTvti); RVOTvti = right ventricular outflow tract velocity time integral; TAPSE = tricuspid annular plane systolic excursion; TR = tricuspid regurgitation; TV = tricuspid valve, TVA = trans tricuspid diastolic velocity, TVa’ = tricuspid annular late diastolic velocity, TVE = transtricuspid early diastolic velocity, TVe’ = tricuspid annular early diastolic velocity, TVs’ = tricuspid annular systolic ejection velocity.

**Table 4:** Right heart dimensions and volumes in 6.5-year-old children born extremely preterm (EXPT) stratified by a neonatal treated patent ductus arteriosus (PDA).

	Accepted for Analysis PDA/No PDA	PDA ^a^ (*n* = 106)	No PDA ^a^ *(n* = 66)	*p*-Value	Adjusted Mean Difference ^b^, (95% CI)	*p*-Value
**RA dimensions**						
RA length	87/57	31.5 (3.1)	32.6 (3.7)	0.054	−0.6 (−1.4;0.3)	0.18
RA width	86/57	27.6 (3.3)	28.4 (2.8)	0.12	−0.7 (−1.7;0.3)	0.18
RA SI	86/57	1.2 (0.1)	1.2 (0.1)	0.94	0.01 (−0.03;0.06)	0.54
**RV dimensions**						
RV length	88/57	50.3 (4.7)	51.2 (4.2)	0.20	−0.4 (−1.6;0.9)	0.57
RV width	88/57	27.0 (2.9)	27.4 (2.6)	0.35	−0.2 (−1.1;0.7)	0.68
RV SI	88/57	1.9 (0.2)	1.9 (0.2)	0.98	0.005 (−0.6;0.07)	0.86
LV/RV length	88/56	1.1 (0.06)	1.1 (0.06)	0.99	0.008 (−0.01;0.03)	0.41
**PA dimensions**						
PV ann	75/50	16.0 (2.3)	16.4 (1.9)	0.44	−0.2 (−0.9;0.4)	0.50
MPA	80/54	16.9 (1.9)	17.2 (1.8)	0.30	−0.2 (−0.8;0.4)	0.47
LPA	70/44	10.2 (2.4)	10.5 (2.0)	0.51	−0.01 (−0.6;0.5)	0.96
RPA	73/44	10.0 (2.2)	10.5 (2.0)	0.30	0.008 (−0.4;0.4)	0.97
**Wall thickness**						
RVAW	57/38	2.7 (0.7)	2.6 (0.8)	0.33	0.09 (−0.2;0.4)	0.55
IVS	90/54	5.5 (0.9)	5.6 (0.9)	0.21	0.007 (−0.3;0.3)	0.96
RWT	55/35	0.32 (0.04)	0.32 (0.05)	0.78	0.003 (−0.02;0.02)	0.77
**Volume**						
SV, ml	66/46	14.8 (2.9)	16.0 (3.6)	0.040	−0.9 (−2.1;0.3)	0.14
CO, l/min	66/46	1.3 (0.3)	1.3 (0.3)	0.68	0.006 (−0.1;0.1)	0.92

Data are expressed in mm if not indicated otherwise; ^a^ Crude value; ^b^ Mean difference adjusted for body surface area (m^2^) and site; CO = cardiac output, IVS = interventricular septae, PA = pulmonary artery, PVann = pulmonary artery valve annulus, MPA = main pulmonary artery, LPA = left pulmonary artery, RA = right atrium, RWT = relative wall thickness, RPA = right pulmonary artery RV = right ventricle, RVAW = right ventricular anterior wall, SI = sphericity index; SV = stroke volume.

**Table 5:** Right heart systolic and diastolic function in 6.5-year-old children born extremely preterm (EXPT) stratified by a neonatal treated patent ductus arteriosus (PDA).

	Accepted for Analysis PDA/No PDA	PDA ^a^ (*n* = 103)	No PDA ^a^ (*n* = 66)	*p*-Value	Adjusted Mean Difference ^b^, (95% CI)	*p*-Value
**Systolic function**						
TAPSE, mm	84/55	20.2 (2.7)	21.4 (2.8)	0.010	−0.8 (−1.7;0.1) *	0.08
TV s’ septal, cm/s	87/49	6.7 (1.1)	6.6 (0.8)	0.36	0.2 (−0.2;0.5)	0.27
TV s’ free wall, cm/s	67/42	11.3 (2.1)	11.6 (2.1)	0.58	−0.2 (−1.0;0.6)	0.69
mpi’, septal	70/37	0.44 (0.07)	0.44 (0.06)	0.49	0.01 (−0.02;0.04)	0.49
mpi’, free wall	79/46	0.37 (0.1)	0.33 (0.1)	0.082	0.03 (0.002;0.06)	0.036
PVR	64/44	0.189 (0.04)	0.170 (0.04)	0.023	0.01 (0.001;0.03)	0.032
TR, m/s	72/47	2.0 (0.3)	2.0 (0.4)	0.82	0.003 (−0.09;0.1)	0.95
RVOTvti, m	83/58	0.12 (0.02)	0.13 (0.03)	0.034	−0.008 (−0.02;−0.001)	0.020
**Diastolic function**						
TVE, cm/s	85/55	49.9 (11.8)	51.9 (10.6)	0.30	−2.0 (−5.5;1.4)	0.25
TVA, cm/s	82/50	32.9 (8.2)	31.7 (7.5)	0.41	0.7 (−2.1;3.5)	0.62
**Septal**						
Annular e’, cm/s	87/49	12.3 (1.5)	12.6 (1.4)	0.15	−0.4 (−0.9;0.1)	0.13
Annular a’, cm/s	87/49	4.7 (1.1)	4.3 (1.0)	0.06	0.4 (0.01;0.8)	0.042
E/e’	80/45	4.1 (1.0)	4.2 (0.9)	0.65	−0.08 (−0.4;0.2)	0.64
ivct, ms	87/49	60 (13)	59 (10)	0.59	1.1 (−3.3;5.5)	0.62
ivrt, ms	87/49	56 (10)	58 (10)	0.29	−1.6 (−4.9;1.8)	0.35
**Free wall**						
TVe’, cm/s	80/47	14.9 (2.8)	15.5 (2.6)	0.27	−0.5 (−1.5;0.4)	0.28
TVa’, cm/s	78/47	7.6 (2.0)	7.3 (1.7)	0.38	0.4 (−0.3;1.0)	0.31
E/e’	74/43	3.4 (0.9)	3.3 (0.7)	0.40	0.1 (−0.2;0.4)	0.39
ivct, ms	79/47	61 (15)	57 (11)	0.058	4.8 (−0.2;9.8)	0.061
ivrt, ms	79/47	35 (21)	35 (22)	0.92	−0.2 (−4.4;4.0)	0.94

^a^ Crude value. Values are presented in mean and SD; ^b^ Mean difference adjusted for site; * Mean difference adjusted for body surface area (m^2^) and site; E/e’= trans tricuspid early diastolic velocity/ tricuspid annular early diastolic velocity; ivct = isovolumic contraction time; ivrt = isovolumic relaxation time; MPI = myocardial performance index; PVR = estimated pulmonary vascular resistance (=TR/RVOTvti); RVOTvti = right ventricular outflow tract velocity time integral; TAPSE = tricuspid annular plane systolic excursion; TR = tricuspid regurgitation; TV = tricuspid valve, TVA = trans tricuspid diastolic velocity, TVa’ = tricuspid annular late diastolic velocity, TVE = transtricuspid early diastolic velocity, TVe’ = tricuspid annular early diastolic velocity, TVs’ = tricuspid annular systolic ejection velocity.

**Table 6:** Right heart dimensions and volumes in 6.5-year-old children born extremely preterm (EXPT) stratified by a neonatal diagnosis of no or moderate bronchopulmonary disease (BPD) versus severe BPD.

	Accepted for Analysis BPD No or Moderate/Severe	BPD ^a^ No or Moderate *n* = 131	BPD ^a^ Severe *n* = 29	*p*-Value	Adjusted Mean Difference ^b^, (95% CI)	*p*-Value
**RA dimensions**						
RA length	113/24	32.2 (3.3)	30.3 (3.4)	0.012	0.18 (−1.33;0.98)	0.76
RA width	112/24	28.0 (3.0)	26.6 (3.2)	0.046	0.49 (−1.83;0.85)	0.47
RA SI	112/24	1.16 (0.12)	1.15 (0.14)	0.72	0.01 (−0.04;0.07)	0.65
**RV dimensions**						
RV length	113/24	50.7 (4.3)	50.3 (5.7)	0.70	1.15 (−0.56;2.86)	0.19
RV width	113/24	27.3 (2.7)	26.2 (3.3)	0.07	1.22 (−2.4;−0.03)	0.044
RV SI	113/24	1.87 (0.19)	1.93 (0.17)	0.13	−0.12 (0.041;0.20)	0.004
LV/RV length	112/24	1.08 (0.06)	1.08 (0.06)	0.81	0.01 (−0.04;0.02)	0.46
**PA dimensions**						
PAV ann	96/24	16.3 (2.1)	15.3 (2.0)	0.024	0.44 (−1.25;0.37)	0.28
MPA	104/23	17.2 (1.8)	16.0 (1.5)	0.005	0.6 (−1.38;0.17)	0.13
LPA	90/17	10.4 (2.3)	9.9 (2.3)	0.44	0.49 (−0.23;1.20)	0.18
RPA	95/18	10.2 (2.1)	9.5 (2.4)	0.20	0.37 (−0.22;0.96)	0.22
**Wall thickness**						
RVAW	75/13	2.7 (0.7)	2.4 (0.6)	0.22	0.2 (−0.7;0.2)	0.26
IVS	116/26	5.6 (0.9)	5.3 (1.0)	0.20	0.002 (−0.4;0.4)	0.99
RWT	72/12	0.32 (0.04)	0.31 (0.05)	0.51	0.01 (−0.04;0.02)	0.49
**Volume**						
SV, ml	85/22	15.4 (3.3)	15.0 (2.8)	0.55	0.02 (−1.6;1.3)	0.80
CO, l/min	84/24	1.33 (0.30)	1.26 (0.23)	0.33	0.05 (−0.19;0.091)	0.49

Data are expressed in mm if not indicated otherwise; ^a^ Crude value; ^b^ Mean difference adjusted for body surface area (m^2^) and site; CO = cardiac output, IVS = interventricular septae, PA = pulmonary artery, PVann = pulmonary artery valve annulus, MPA = main pulmonary artery, LPA = left pulmonary artery, RA = right atrium, RWT = relative wall thickness, RPA = right pulmonary artery RV = right ventricle, RVAW =right ventricular anterior wall, SI = spericity index; SV = stroke volume.

**Table 7:** Right heart systolic and diastolic function in 6.5-year-old children born extremely preterm (EXPT) stratified by neonatal diagnosis of no or moderate bronchopulmonary disease (BPD) versus severe BPD.

	Accepted for Analysis BPD No or Moderate/Severe	BPD ^a^ No or Moderate *n* = 131	BPD ^a^ Severe *n* = 29	*p*-Value	Adjusted Mean Difference ^b^, (95% CI)	*p*-Value
**Systolic function**						
TAPSE, mm	107/24	20.7 (2.7)	19.8 (3.0)	0.15	0.3 (−1.5;0.8) *	0.59
TVs’ septal, cm/s	106/23	6.6 (1.1)	6.8 (1.0)	0.57	−0.1 (−0.6;0.4)	0.68
TVs’ free wall, cm/s	83/20	11.4 (2.1)	11.7 (2.3)	0.50	0.2 (−0.8;1.3)	0.64
mpi’, septal	81/19	0.44 (0.07)	0.44 (0.05)	0.97	0.003 (−0.03;0.04)	0.88
mpi’, free wall	97/20	0.35 (0.11)	0.41 (0.14)	0.025	0.02 (−0.03;0.06)	0.45
PVR	83/17	0.182 (0.04)	0.171 (0.04)	0.33	0.003 (−0.01;0.02)	0.76
TR, m/s	92/19	2.0 (0.3)	2.0 (0.3)	0.75	0.1 (−0.004;0.3)	0.84
RVOTvti, m	109/24	11.8 (2.4)	12.6 (2.3)	0.20	0.04 (−0.9;0.9)	0.94
**Diastolic function**						
TVE, cm/s	108/24	50.9 (11.9)	50.8 (9.3)	0.95	−3.6 (−8.3;1.0)	0.12
TVA, cm/s	103/22	32.5 (7.9)	33.9 (8.6)	0.46	−0.7 (−3.1;4.4)	0.72
**Septal**						
TVe’, cm/s	106/23	12.4 (1.5)	12.3 (1.3)	0.63	−0.4 (−1.1;0.3)	0.25
TVa’, cm/s	106/23	4.6 (1.0)	4.6 (1.4)	0.99	−0.1 (−0.6;0.4)	0.65
E/e’	97/21	4.2 (0.8)	4.1 (1.0)	0.93	−0.3 (−0.7;0.2)	0.21
ivct, ms	106/23	60 (13)	56 (10)	0.14	−3.7 (−9.6;2.1)	0.21
ivrt, ms	106/23	57 (10)	60 (7)	0.13	2.2 (−2.2;6.6)	0.32
**Free wall**						
TVe’, cm/s	99/20	15.1 (2.6)	15.5 (3.6)	0.62	−0.1 (−1.4;1.2)	0.87
TVa’, cm/s	97/20	7.5 (1.8)	7.6 (2.4)	0.87	−0.1 (−1.1;0.8)	0.78
E/e’	91/18	3.4 (0.8)	3.3 (0.6)	0.60	0.3 (−0.7;0.2)	0.21
ivct, ms	98/20	59 (14)	62 (14)	0.38	2.9 (−4.1;9.8)	0.42
ivrt, ms	98/20	34 (21)	46 (23)	0.017	−1.9 (−3.9;7.8)	0.52

^a^ Crude value. Values are presented in mean and SD; ^b^ Mean difference adjusted for site; * Mean difference adjusted for body surface area (m^2^) and site; E/e’ = trans tricuspid early diastolic velocity/ tricuspid annular early diastolic velocity; ivct = isovolumic contraction time; ivrt = isovolumic relaxation time; MPI = myocardial performance index; PVR = estimated pulmonary vascular resistance (=TR/RVOTvti); RVOTvti = right ventricular outflow tract velocity time integral; TAPSE = tricuspid annular plane systolic excursion; TR = tricuspid regurgitation; TV = tricuspid valve, TVA = trans tricuspid diastolic velocity, TVa’ = tricuspid annular late diastolic velocity, TVE = transtricuspid early diastolic velocity, TVe’ = tricuspid annular early diastolic velocity, TVs’ = tricuspid annular systolic ejection velocity.

## Supplementary Materials

The following are available online at https://www.mdpi.com/2077-0383/10/1/122/s1, Table S1: Right heart dimensions and volumes in 6.5-year-old children born extremely preterm, stratified by small-for-gestational age (SGA) & appropriate-for-gestational age (AGA), Table S2: Right heart systolic and diastolic function in 6.5-year-old children born extremely preterm, stratified by small-for-gestational age (SGA) & appropriate-for-gestational age (AGA), Table S3: Right heart dimensions and volumes in 6.5-year-old children born extremely preterm stratified by gestational week 22–24 (GW22–24) & 25–26 postmenstrual age (GW25–26), Table S4: Right heart systolic and diastolic function in 6.5-year-old children born extremely preterm stratified by gestational week 22–24 (GW22–24) & 25–26 (GW25–26) postmenstrual age, Table S5: Right heart dimensions and volumes in 6.5-year-old children born extremely preterm (EXP) and in controls born at term (CTRL) stratified by girls, Table S6: Right heart systolic and diastolic function in 6.5-year-old children born extremely preterm (EXP) and in controls born at term (CTRL) stratified by girls, Table S7: Right heart dimensions and volumes in 6.5-year-old children born extremely preterm (EXP) and in controls born at term (CTRL) stratified by boys, Table 8: Right heart systolic and diastolic function in 6.5-year-old children born extremely pre-term (EXP) and in controls born at term (CTRL) stratified by boys.
