# Supplementary material for: Right Heart Structure, Geometry and Function Assessed by Echocardiography in 6-Year-Old Children Born Extremely Preterm—A Population-Based Cohort Study
Source: J Clin Med. 2020 Dec 31;10(1):122. doi: 10.3390/jcm10010122 (PMC7795537; doi:10.3390/jcm10010122)
Supplement: Supplementary file 1 [file jcm-10-00122-s001.pdf]

Supplement tables

Supplemental table 1. Right heart dimensions and volumes in 6.5-year-old children born extremely preterm, stratified by small-for-gestational age (SGA) & appropriate-for-gestational age (AGA).

	Accepted for analysis	SGA[a] (n=27)	AGA[a] (n=142)	p-value	Adjusted mean difference[b] (95% CI)	p-value
RA dimensions						
RA length	22/122	31.1(4.3)	32.0(3.2)	0.24	0.7(-0.5;1.9)	0.25
RA width	22/122	26.8(3.1)	28.1(3.1)	0.09	-0.2(-1.6;1.1)	0.74
RA SI	22/121	1.2(0.1)	1.2(0.1)	0.63	0.03(-0.03;0.09)	0.31
RV dimensions						
RV length	21/124	48.1(4.7)	51.1(4.4)	0.005	-1.5(-3.2;0.3)	0.10
RV width	21/124	27.4(3.4)	27.1(2.7)	0.63	0.4(-0.8;1.7)	0.52
RV SI	21/124	1.8(0.2)	1.9(0.2)	0.004	-0.08(-0.2;0.006)	0.07
LV/RV length	21/123	1.1(0.7)	1.1(0.6)	0.47	0.002(-0.2;0.03)	0.89
PA dimensions						
PV ann	18/107	15.2(1.8)	16.3(2.1)	0.037	-0.4(-1.3;0.5)	0.43
MPA	18/116	16.1(1.8)	17.2(1.8)	0.024	-0.5(-1.3;0.3)	0.23
LPA	19/95	9.7(2.4)	10.5(2.2)	0.15	0.2(-0.5;0.9)	0.62
RPA	19/101	9.7(2.4)	10.3(2.1)	0.23	0.4(-0.2;1.0)	0.22
Wall thickness						
RVAW	14/81	2.8(1.0)	2.6(0.7)	0.44	0.3(-0.2;0.7)	0.22
IVS	24/125	5.4(1.0)	5.6(0.9)	0.41	0.05(-0.3;0.4)	0.78
RWT	12/78	0.31(0.04)	0.32(0.04)	0.37	-0.01(-0.04;0.02)	0.42
Volumes						
SV, ml	17/95	15.6(4.2)	15.2(3.0)	0.62	0.3(-1.3;2.0)	0.70
CO, l/min	17/94	1.28(0.26)	1.32(0.30)	0.57	-0.05(-0.21;0.10)	0.52

Data are expressed in mm if not indicated otherwise.

[a] Crude value.

[b] Mean difference adjusted for body surface area (m^2) and site.

CO= cardiac output, IVS= interventricular septae, PA= pulmonary artery, PVann= pulmonary artery valve annulus, MPA= main pulmonary artery, LPA= left pulmonary artery, RA= right atrium, RWT= relative wall thickness, RPA= right pulmonary artery RV= right ventricle, RVAW=right ventricular anterior wall, SI=sphericity index; SV=stroke volume.

Supplemental table 2. Right heart systolic and diastolic function in 6.5-year-old children born extremely preterm, stratified by small-for-gestational age (SGA) & appropriate-for-gestational age (AGA).

	Accepted for analysis	SGA[a] (n=27)	AGA[a] (n=142)	p-value	Adjusted mean difference[a], (95% CI)	p-value
Systolic function						
TAPSE, mm	23/116	19.7(2.7)	20.8(2.8)	0.09	-0.6(-1.8;0.6)	0.34
TVs' septal, cm/s	19/117	6.4(0.8)	6.7(1.0)	0.31	-0.5(-1.0;0.001)	0.051
TV s' free wall, cm/s	15/94	12.1(3.2)	11.3(1.9)	0.21	0.5(-0.6;1.7)	0.39
mpi', septal	15/92	0.46(0.06)	0.44(0.07)	0.25	0.02(-0.01;0.06)	0.23
mpi', free wall	18/107	0.39(0.1)	0.35(0.1)	0.17	0.01(-0.03;0.06)	0.59
PVR, TR/RVOTvti	17/91	0.176(0.05)	0.182(0.04)	0.58	0.01(-0.008;0.03)	0.28
TR, m/s	17/102	1.96(0.34)	2.04(0.29)	0.27	-0.02(-0.2;0.1)	0.75
RVOTvti, m	23/118	0.13(0.03)	0.12(0.02)	0.13	0.001(-0.008;0.01)	0.80
Diastolic function						
TVE, cm/s	22/118	51.1(10.9)	50.6(11.4)	0.84	-2.6(-7.3;2.1)	0.27
TVA, cm/s	21/111	32.8(8.4)	32.4(7.9)	0.84	-0.3(-4.0;3.5)	0.88
Septal						
Annular e', cm/s	19/117	12.3(1.3)	12.4(1.5)	0.73	-0.3(-1.0;0.4)	0.40
Annular a', cm/s	19/117	4.4(1.0)	4.6(1.1)	0.62	-0.2(-0.8;0.3)	0.36
E/e'	18/107	4.2(1.1)	4.1(0.9)	0.55	-0.1(-0.6;0.3)	0.59
ivct, msec	19/117	63(12)	59(12)	0.23	4.5(-1.6;10.6)	0.15
ivrt, msec	19/117	60(7)	57(10)	0.22	1.6(-3.1;6.4)	0.50
Free wall						
Tve', cm/s	18/109	16.4(4.0)	15.0(2.4)	0.043	1.1(-0.2;2.4)	0.10
TVa', cm/s	18/107	8.4(2.0)	7.4(1.9)	0.027	0.9(-0.04;1.9)	0.059
E/e'	17/100	3.3(0.9)	3.4(0.8)	0.76	-0.2(-0.6;0.2)	0.39
ivct, msec	18/108	59(10)	60(14)	0.84	-0.8(-7.9;6.2)	0.82
ivrt, msec	18/108	41(24)	34(20)	0.20	1.2(-4.7;7.0)	0.70

[a] Crude value. Values are presented in mean and SD.

[b] Mean difference adjusted for site.

* Mean difference adjusted for body surface area (m^2) and site.

E/e'= early emptying blood velocity/ early emptying tissue velocity; ivct= isovolumic contraction time; ivrt=isovolumic relaxation time; MPI=myocardial performance index; PVR= estimated pulmonary vascular resistance (=TR/RVOTvti); RVOTvti= right ventricular outflow tract velocity time integral; TAPSE=tricuspid annular plane systolic excursion; TR=tricuspid regurgitation; TV=tricuspid valve, TVA= trans tricuspid diastolic velocity, TVa'= tricuspid annular late diastolic velocity, TVE= transtricuspid early diastolic velocity, TVe'= tricuspid annular early diastolic velocity, TVs'= tricuspid annular systolic ejection velocity.

Supplemental table 3. Right heart dimensions and volumes in 6.5-year-old children born extremely preterm stratified by gestational week 22-24 (GW22-24) & 25-26 postmenstrual age (GW25-26).

	Accepted for analysis	GW 22-24[a] (n=50)	GW 25-26[a] (n=119)	p-value	Adjusted mean difference[b](95% CI)	p-value
RA dimensions						
RA length	40/104	30.8(3.1)	32.4(3.4)	0.018	0.2(-0.7;1.2)	0.61
RA width	40/103	27.2(3.3)	28.2(3.0)	0.08	0.3(-0.8;1.4)	0.63
RA SI	40/103	1.14(0.1)	1.16(0.1)	0.64	-0.001(-0.05;0.05)	0.97
RV dimensions						
RV length	42/103	50.2(4.7)	50.8(4.4)	0.46	-1.2(-2.5;0.2)	0.09
RV width	42/103	26.6(3.5)	27.3(2.4)	0.16	-0.3(-0.7;1.3)	0.55
RV SI	42/103	1.90(0.2)	1.87(0.2)	0.23	0.07(-0.1;-0.004)	0.038
LV/RV length	42/102	1.08(0.05)	1.08(0.07)	0.95	0.01(-0.008;0.03)	0.23
PA dimensions						
PV ann	33/92	16.4(1.8)	17.2(1.8)	0.039	0.4(-0.2;1.1)	0.20
MPA	33/101	16.4(1.8)	17.2(1.8)	0.037	0.4(-0.2;1.1)	0.20
LPA	27/87	10.3(2.4)	10.4(2.3)	0.92	0.2(-0.4;0.8)	0.55
RPA	29/91	9.8(2.0)	10.3(2.2)	0.26	0.4(-0.2;0.8)	0.17
Wall thickness						
RVAW	24/71	2.6(0.6)	2.7(0.8)	0.89	0.03(-0.3;0.4)	0.85
IVS	41/108	5.4(1.0)	5.6(0.8)	0.26	-0.08(-0.4;0.2)	0.59
RWT	24/66	0.32(0.06)	0.32(0.04)	0.75	-0.005(-0.03;0.02)	0.69
Volumes						
SV, ml	30/82	14.8(3.4)	15.4(3.2)	0.38	-0.2(-1.1;1.6)	0.76
CO, l/min	29/82	1.28(0.35)	1.33(0.27)	0.44	-0.02(-0.1;0.2)	0.71

Data are expressed in mm if not indicated otherwise.

[a] Crude value.

[b] Mean difference adjusted for body surface area (m^2) and site.

CO= cardiac output, IVS= interventricular septae, PA= pulmonary artery, PVann= pulmonary artery valve annulus, MPA= main pulmonary artery, LPA= left pulmonary artery, RA= right atrium, RWT= relative wall thickness, RPA= right pulmonary artery RV= right ventricle, RVAW=right ventricular anterior wall, SI=spericity index; SV=stroke volume.

Supplemental table 4. Right heart systolic and diastolic function in 6.5-year-old children born extremely preterm stratified by gestational week 22-24 (GW22-24) & 25-26 (GW25-26) postmenstrual age.

	Accepted for analysis	GW 22-24[a] (n=50)	GW 25-26[a] (n=119)	p-value	Adjusted mean difference[b], (95% CI)	p-value
Systolic function						
TAPSE, mm	40/99	19.9(2.5)	21.0(2.9)	0.048	-0.4(-0.5;1.4)	0.36
TV septal S', cm/s	38/98	6.4(0.8)	6.8(1.1)	0.036	-0.4(0.08;0.8)	0.017
TV lateral S', cm/s	31/78	11.5(1.7)	11.4(2.3)	0.86	0.1(-1.0;0.8)	0.81
mpi', septal	30/77	0.44(0.08)	0.44(0.06)	0.63	0.007(-0.02;0.04)	0.63
mpi', free wall	33/92	0.38(0.1)	0.35(0.1)	0.19	0.02(-0.05;0.01)	0.26
PVR	32/76	0.185(0.04)	0.179(0.04)	0.50	0.003(-0.02;0.01)	0.67
TR, m/s	34/85	2.05(0.2)	2.02(0.3)	0.59	0.01(-0.1;0.09)	0.80
RVOTvti, m	41/100	0.12(0.02)	0.12(0.02)	0.90	0.0009(-0.006;0.008)	0.82
Diastolic function						
TVE, cm/s	39/101	50.0(10.6)	50.9(11.6)	0.66	-1.2(-2.6;4.9)	0.53
TVA, cm/s	35/97	31.6(7.8)	32.7(8.0)	0.48	-1.4(-1.6;4.4)	0.36
Septal						
Annular e', cm/s	38/98	12.5(1.4)	12.4(1.5)	0.69	-0.09(-0.6;0.4)	0.75
Annular a', cm/s	38/98	4.2(1.1)	4.6(1.0)	0.041	-0.4(0.04;0.8)	0.032
E/e'	34/91	4.0(0.9)	4.2(1.0)	0.34	0.2(-0.2;0.5)	0.28
ivct, msec	38/98	60(13)	60(12)	0.91	-0.4(-5.0;4.3)	0.88
ivrt, msec	38/98	56(9)	58(10)	0.38	-1.9(-1.7;5.5)	0.30
Free wall						
TVe´, cm/s	34/93	15.3(2.1)	15.1(2.9)	0.70	0.2(-1.2;0.9)	0.78
TVa', cm/s	32/93	7.7(2.0)	7.4(1.9)	0.55	0.2(-1.0;0.6)	0.58
E/e'	31/86	3.2(0.8)	3.4(0.8)	0.29	-0.2(-0.1;0.5)	0.27
ivct, msec	33/93	60(16)	60(13)	0.86	-0.4(-6.0;5.1)	0.88
ivrt, msec	33/93	39(21)	34(21)	0.25	2.7(-7.3;1.8)	0.24

[a] Crude value. Values are presented in mean and SD.

[b] Mean difference adjusted for site.

* Mean difference adjusted for body surface area (m^2) and site.

E/e'= early emptying blood velocity/ early emptying tissue velocity; ivct= isovolumic contraction time; ivrt=isovolumic relaxation time; MPI=myocardial performance index; PVR= estimated pulmonary vascular resistance (=TR/RVOTvti); RVOTvti= right ventricular outflow tract velocity time integral; TAPSE=tricuspid annular plane systolic excursion; TR=tricuspid regurgitation; TV=tricuspid valve, TVA= trans tricuspid diastolic velocity, TVa'= tricuspid annular late diastolic velocity, TVE= transtricuspid early diastolic velocity, TVe'= tricuspid annular early diastolic velocity, TVs'= tricuspid annular systolic ejection velocity.

Supplemental table 5. Right heart dimensions and volumes in 6.5-year-old children born extremely preterm (EXP) and in controls born at term (CTRL) stratified by girls.

	Accepted for analysis	Girls EXP[a] (n=78)	Girls CTRL[a] (n=55)	p-value	Adjusted mean difference[b,] (95% CI)	p-value
RA dimensions						
RA length	67/50	31.9(3.2)	34.8(3.1)	<0.001	-0.7(-1.8;0.4)	0.23
RA width	67/50	27.2(3.0)	30.1(2.4)	<0.001	-1.5(-2.6;-0.5)	0.004
RA SI	67/50	1.2(0.1)	1.2(0.1)	0.32	0.04(-0.004;0.9)	0.07
RV dimensions						
RV length	68/52	51.0(4.1)	53.2(4.7)	0.007	-0.4(-2.0;1.2)	0.65
RV width	68/52	27.3(2.9)	28.3(2.4)	0.036	-0.8(-1.9;0.2)	0.11
RV SI	68/52	1.9(0.2)	1.9(0.2)	0.86	0.05(-0.03;0.1)	0.23
LV/RV length	68/52	1.07(0.06)	1.10(0.06)	0.005	-0.03(-0.05;-0.006)	0.016
PA dimensions						
PV ann	55/33	15.8(2.0)	17.4(1.9)	0.0003	-0.6(-1.5;0.2)	0.13
MPA	60/50	16.9(1.6)	17.8(1.8)	0.006	-0.3(-0.9;0.4)	0.46
LPA	52/46	10.1(2.1)	11.6(1.3)	0.0001	-0.2(-0.8;0.4)	0.47
RPA	53/48	10.0(2.0)	11.3(1.3)	0.0001	0.04(-0.5;0.6)	0.87
Wall thickness						
RVAW	42/47	2.7(0.8)	2.7(0.6)	0.92	0.02(-0.3;0.4)	0.88
IVS	67/54	5.5(0.9)	5.9(0.9)	0.005	-0.2(-0.5;0.2)	0.35
RWT	41/45	0.31(0.04)	0.30(0.05)	0.50	0.008(-0.01;0.03)	0.50
Volumes						
SV, ml	49/33	14.7(3.0)	17.2(2.8)	0.0002	-2.1(-3.6;-0.6)	0.006
CO, l/min	48/31	1.3(0.3)	1.4(0.2)	0.013	-0.1(-0.3;0.01)	0.070

Data are expressed in mm if not indicated otherwise.

[a] Crude value.

[b] Mean difference adjusted for body surface area (m^2) and site.

CO= cardiac output, IVS= interventricular septae, PA= pulmonary artery, PVann= pulmonary artery valve annulus, MPA= main pulmonary artery, LPA= left pulmonary artery, RA= right atrium, RWT= relative wall thickness, RPA= right pulmonary artery RV= right ventricle, RVAW=right ventricular anterior wall, SI=spericity index; SV=stroke volume.

Supplementalal table 6. Right heart systolic and diastolic function in 6.5-year-old children born extremely preterm (EXP) and in controls born at term (CTRL) stratified by girls.

	Accepted for analysis	Girls EXP[a] (n=78)	Girls CTRL[a] (n=55)	p-value	Adjusted mean difference[b],(95% CI)	p-value
Systolic function						
TAPSE, mm	65/52	20.1(2.5)	20.6(2.3)	0.26	0.09(-0.9;1.1)*	0.86
TVs' septal, cm/s	63/45	6.6(1.1)	6.5(0.8)	0.36	-0.08(-0.5;0.3)	0.69
TVs' free wall, cm/s	49/28	11.1(1.9)	11.8(1.7)	0.15	-1.1(-2.1;-0.2)	0.019
mpi', septal	53/33	0.44(0.06)	0.45(0.06)	0.67	-0.007(-0.04;0.02)	0.64
mpi', free wall	59/45	0.37(0.1)	0.32(0.08)	0.020	-0.004(-0.04;0.03)	0.84
PVR	51/48	0.182(0.04)	0.172 (0.3)	0.21	0.02(0.007;0.03)	0.003
TR, m/s	56/49	2.0(0.3)	2.0(0.1)	0.70	0.09(0.008;0.2)	0.032
RVOTvti, cm	65/53	11.6(1.9)	12.0(2.0)	0.24	-0.9(-1.6;-0.2)	0.014
Diastolic function						
TVE, cm/s	64/53	49.2(11.5)	47.9(10.1)	0.51	-1.5(-5.4;2.5)	0.45
TVA, cm/s	60/51	31.6(7.6)	31.6(7.8)	0.99	-1.0(-4.0;2.1)	0.54
Septal						
Annular e', cm/s	63/45	12.3(1.7)	11.9(1.4)	0.12	0.2(-0.4;0.9)	0.47
Annular a', cm/s	63/45	4.4(1.0)	4.4(1.0)	0.96	-0.2(-0.6;0.3)	0.48
E/e'	57/45	4.0(1.0)	4.0(1.0)	0.64	-0.2(-0.6;0.2)	0.42
ivct, msec	63/45	62(11)	65(12)	0.16	-2.0(-7.0;3.0)	0.43
ivrt, msec	63/44	57(10)	57(9)	0.79	-0.9(-4.5;2.8)	0.64
Free wall						
TVe´, cm/s	61/45	14.7(2.4)	14.1(1.9)	0.16	-0.2(-1.1;0.6)	0.61
TVa', cm/s	60/45	7.4(1.8)	6.8(1.9)	0.07	0.4(-0.4;1.2)	0.32
E/e'	55/45	3.4(0.8)	3.4(0.9)	0.93	-0.09(-0.5;0.3)	0.62
ivct, msec	60/45	60(14)	64(15)	0.14	-3.9(-10.2;2.2)	0.21
ivrt, msec	60/45	38(22)	23(11)	<0.001	2.0(-2.4;6.1)	0.37

[a] Crude value. Values are presented in mean and SD.

[b] Mean difference adjusted for site.

* Mean difference adjusted for body surface area (m^2) and site.

E/e'= early emptying blood velocity/ early emptying tissue velocity; ivct= isovolumic contraction time; ivrt=isovolumic relaxation time; MPI=myocardial performance index; PVR= estimated pulmonary vascular resistance (=TR/RVOTvti); RVOTvti= right ventricular outflow tract velocity time integral; TAPSE=tricuspid annular plane systolic excursion; TR=tricuspid regurgitation; TV=tricuspid valve, TVA= trans tricuspid diastolic velocity, TVa'= tricuspid annular late diastolic velocity, TVE= transtricuspid early diastolic velocity, TVe'= tricuspid annular early diastolic velocity, TVs'= tricuspid annular systolic ejection velocity.

Supplemental table 7. Right heart dimensions and volumes in 6.5-year-old children born extremely preterm (EXP) and in controls born at term (CTRL) stratified by boys.

	Accepted for analysis	Boys EXP[a] (n=90)	Boys CTRL[a] (n=79)	p-value	Adjusted mean difference[b] , (95% CI)	p-value
RA dimensions						
RA length	84/76	32.0(3.5)	36.2(3.6)	<0.001	-1.3(-2.4;-0.3)	0.014
RA width	83/76	28.5(3.1)	32.4(3.3)	<0.001	-2.4(-3.5;-1.2)	<0.001
RA SI	83/76	1.1(0.1)	1.1(0.1)	0.69	0.04(-0.005;0.09)	0.08
RV dimensions						
RV length	84/76	50.5(4.8)	53.4(3.6)	<0.001	-0.5(-1.9;0.9)	0.49
RV width	84/77	27.2(2.8)	30.2(2.8)	<0.001	-2.2(-3.2;-1.2)	<0.001
RV SI	84/76	1.9(0.2)	1.8(0.2)	0.008	0.1(0.05;0.2)	0.001
LV/RV length	83/75	1.1(0.07)	1.1(0.06)	0.18	-0.02(-0.04;0.003)	0.08
PA dimensions						
PV ann	75/57	16.5(2.3)	17.9(1.7)	0.0002	-0.001(-0.7;0.7)	1.00
MPA	81/75	17.1(2.0)	18.1(1.8)	0.002	0.2(-0.5;0.8)	0.62
LPA	68/66	10.6(2.4)	12.3(1.4)	<0.001	-0.2(-0.7;0.4)	0.59
RPA	72/70	10.4(2.2)	12.2(1.4)	<0.001	-0.06(-0.5;0.4)	0.80
Wall thickness						
RVAW	58/65	2.6(0.7)	2.7(0.8)	0.49	-0.02(-0.3;0.3)	0.92
IVS	89/78	5.6(0.9)	6.2(0.7)	<0.001	-0.04(-0.3;0.2)	0.77
RWT	54/64	0.32(0.04)	0.30(0.04)	0.006	0.02(0.004;0.04)	0.019
Volumes						
SV, ml	68/52	15.6(3.3)	16.8(2.6)	0.041	-0.4(-1.6;0.9)	0.54
CO, l/min	67/52	1.3(0.3)	1.4(0.2)	0.20	-0.03(-0.2;0.09)	0.61

Data are expressed in mm if not indicated otherwise.

[a] Crude value.

[b] Mean difference adjusted for body surface area (m^2) and site.

CO= cardiac output, IVS= interventricular septae, PA= pulmonary artery, PVann= pulmonary artery valve annulus, MPA= main pulmonary artery, LPA= left pulmonary artery, RA= right atrium, RWT= relative wall thickness, RPA= right pulmonary artery RV= right ventricle, RVAW=right ventricular anterior wall, SI=spericity index; SV=stroke volume.

Supplemental table 8. Right heart systolic and diastolic function in 6.5-year-old children born extremely preterm (EXP) and in controls born at term (CTRL) stratified by boys.

	Accepted for analysis	Boys EXP[a] (n=90)	Boys CTRL[a] (n=79)	p-value	Adjusted mean difference[b] ,(95% CI)	p-value
Systolic function						
TAPSE, mm	81/71	21.1(3.1)	21.5(2.9)	0.36	1.32(0.3;2.4)*	0.013
TVs' septal, cm/s	80/69	6.7(1.0)	6.7(0.7)	0.97	-0.2(-0.4;0.1)	0.29
TVs' free wall, cm/s	66/49	11.6(2.4)	11.9(1.8)	0.52	-0.2(-1.0;0.7)	0.69
mpi', septal	61/50	0.43(0.07)	0.45(0.07)	0.12	-0.02(-0.05;0.01)	0.23
mpi', free wall	73/65	0.34(0.1)	0.34(0.1)	0.80	-0.04(-0.08;-0.01)	0.009
PVR	63/69	0.183(0.04)	0.176(0.03)	0.22	0.02(0.006;0.03)	0.002
TR, m/s	69/72	2.1(0.2)	2.0(0.1)	0.036	0.10(0.03;0.2)	0.005
RVOTvti, cm	83/73	12.1(2.7)	11.6(1.5)	0.17	-0.3(-0.9;0.3)	0.36
Diastolic function						
TVE, cm/s	83/76	51.4(10.9)	49.9(10.4)	0.36	-0.5(-3.6;2.5)	0.73
TVA, cm/s	79/75	33.3(7.9)	32.3(8.0)	0.43	0.004(-2.7;2.7)	1.00
Septal						
Annular e', cm/s	80/68	12.4(1.2)	12.2(1.3)	0.51	-0.02(-0.5;0.4)	0.95
Annular a', cm/s	80/69	4.6(1.1)	4.5(0.8)	0.31	0.09(-0.2;0.4)	0.59
E/e'	75/67	4.1(0.9)	4.0(0.9)	0.44	-0.04(-0.3;0.2)	0.78
ivct, msec	80/68	58(12)	65(13)	0.0009	-6.5(-11.0;-2.1)	0.004
ivrt, msec	80/68	57(10)	56(8)	0.75	-0.5(-3.5;2.4)	0.73
Free wall						
TVe´, cm/s	73/68	15.4(3.0)	14.1(2.2)	0.006	1.1(0.9;1.9)	0.032
TVa', cm/s	72/68	7.7(2.0)	6.9(1.6)	0.016	0.6(-0.09;1.2)	0.09
E/e'	69/67	3.4(0.8)	3.6(0.9)	0.17	-0.3(-0.6;-0.02)	0.038
ivct, msec	73/68	59(14)	66(16)	0.007	-7.4(-12.7;-2.2)	0.006
ivrt, msec	73/66	32(21)	26(14)	0.038	-2.5(-7.2;2.3)	0.30

[a] Crude value. Values are presented in mean and SD.

[b] Mean difference adjusted for site.

* Mean difference adjusted for body surface area (m^2) and site.

E/e'= early emptying blood velocity/ early emptying tissue velocity; ivct= isovolumic contraction time; ivrt=isovolumic relaxation time; MPI=myocardial performance index; PVR= estimated pulmonary vascular resistance (=TR/RVOTvti); RVOTvti= right ventricular outflow tract velocity time integral; TAPSE=tricuspid annular plane systolic excursion; TR=tricuspid regurgitation; TV=tricuspid valve, TVA= trans tricuspid diastolic velocity, TVa'= tricuspid annular late diastolic velocity, TVE= transtricuspid

early diastolic velocity, TVe'= tricuspid annular early diastolic velocity, TVs'= tricuspid annular systolic ejection velocity.
